# Therapeutic efficacy of traditional Chinese medicine, Shen-Mai San, in cancer patients undergoing chemotherapy or radiotherapy: study protocol for a randomized, double-blind, placebo-controlled trial

**DOI:** 10.1186/1745-6215-13-232

**Published:** 2012-12-03

**Authors:** Lun-Chien Lo, Chia-Yun Chen, Shou-Tung Chen, Hung-Chang Chen, Tsung-Chieh Lee, Cheng-Shyong Chang

**Affiliations:** 1 Department of Chinese Medicine, Changhua Christian Hospital, 135 Nanxiao St, Changhua City, Changhua Country, 500, Taiwan; 2 Department of Mathematics and Institute of Statistics and Information Science, National Changhua University of Education, No.1, Jin-De Road, Changhua City, Changhua Country, 500, Taiwan; 3 Comprehensive Breast Cancer Center, Changhua Christian Hospital, 135 Nanxiao St, Changhua City, Changhua Country, 500, Taiwan; 4 Division of General Surgery, Department of Surgery, Changhua Christian Hospital, 135 Nanxiao St, Changhua City, Changhua Country, 500, Taiwan; 5 Division of Colorectal Surgery, Department of Surgery, Changhua Christian Hospital, 135 Nanxiao St, Changhua City, Changhua Country, 500, Taiwan; 6 Department of Hematology and Oncology, Changhua Christian Hospital, 135 Nanxiao St, Changhua City, Changhua Country, 500, Taiwan

**Keywords:** Traditional Chinese Medicine, Cancer, Chemotherapy, Radiotherapy

## Abstract

**Background:**

Cancer is one of the major health issues worldwide. An increasing number of cancer patients are offered treatment with surgery, chemotherapy and radiotherapy. Traditional Chinese medicine (TCM) is one of the most common complementary therapies offered to cancer patients in Taiwan. We designed a randomized, double-blind, placebo-controlled clinical trial to evaluate the efficacy of TCM in patients with cancer.

**Methods/design:**

In this study, inclusion criteria are postoperative patients with histologically confirmed cancer within 3 years who are undergoing chemotherapy or radiotherapy, more than 18 years old, have given signed informed consent, have the ability to read Chinese, and the ability for oral intake.

Exclusion criteria include being pregnant, breast feeding, having completed chemotherapy or radiotherapy, brain metastasis with Eastern Cooperative Oncology Group (ECOG) performance status of two to four, delusion or hallucinations, acute infection, and have received medications under other clinical trials.

The patients were separated into an intervention group (Shen-Mai-San, SMS) and a placebo group for four weeks using a randomized, double-blind procedure. The European Organization for Research and Treatment of Cancer (EORTC) Quality of Life questionnaire (QOL-C30) was used to evaluate the quality of life. General data, hemoglobin (Hb), hematocrit (Hct), glutamic-oxalacetic transaminase (GOT), glutamic-pyruvic transaminase (GPT), blood urea nitrogen (BUN), creatinine, carcinoembryonic antigen (CEA), TCM diagnosis data and heart rate variability (HRV) were also recorded. These data were collected at baseline, two weeks and four weeks after receiving medication. The patients were prescribed granules which contained therapeutic medicines or placebo. Paired-T test was used for statistical analysis.

**Discussion:**

Shen-Mai-San is composed of processed *Ginseng radis, Liriope spicata, and Schizandrae fructus*. It was found to be effective for treating cancer-related fatigue and had anti-fatigue activity. In TCM theory, SMS has a synergistic effect for qi and yin deficiency and has the ability to prevent fatigue. The symptoms of qi and yin deficiency are similar to chemotherapy- or radiotherapy-induced side effects. In order to evaluate the efficacy of SMS on cancer treatment, we designed a randomized, double-blind, placebo-controlled trial.

**Trial registration:**

This study is registered to Clinical Trails.gov NCT01580358

## Background

Cancer has been the most horrible disease worldwide. In Taiwan, cancer has been ranked as the leading cause of death since 1982 [1]. Liver cancer, lung cancer and colon cancer are the leading cancers and account for 50% of all cancers in Taiwan [1]. The treatment of cancer includes surgery, chemotherapy, radiotherapy, target therapy, and so on. The choice of management depends on cancer type and stage. Treatment generally has a beneficial effect on curbing the progression of the disease, but sometimes causes disturbing side effects, such as nausea, vomiting [2], mucositis [3], general weakness [4] and so on which may decrease a patient’s quality of life [5]. The use of complementary and alternative medicine (CAM) has increased over the past years around the world [6]. Some studies indicate that the use of CAM is found more frequently in patients with cancer than in the general population and the use of CAM is common in these patients [7,8]. The use of CAM increased among these patients who had a lower quality of life [9]. Many studies indicate an improvement in the quality of life and general health in cancer patients adopting CAM treatment [10,11]. CAM treatment includes dietary supplements, prayers, traditional Chinese herbs and botanicals [12].

Traditional Chinese medicine (TCM) is one of the most common CAM used in Taiwan. Some cancer patients search for a second opinion or TCM instead of regular treatment. Many patients in Taiwan turn to TCM due to poor quality of life or general weakness after surgery, radiotherapy or chemotherapy. Some studies reported that TCM did demonstrate some beneficial effects on maintaining immune function and liver protection in patients who were simultaneously receiving chemotherapy [13,14]. The purpose of this study is to evaluate the efficacy of TCM in improving the quality of life of cancer patients undergoing chemotherapy or radiotherapy. Although many CAMs have demonstrated positive effects on lessening the side effects suffered after chemotherapy or radiotherapy, a rigid validation using a randomized controlled trial (RCT) remains the best way to examine the effect of TCM in these patients.

## Methods/Design

### Design

**Figure 1:**
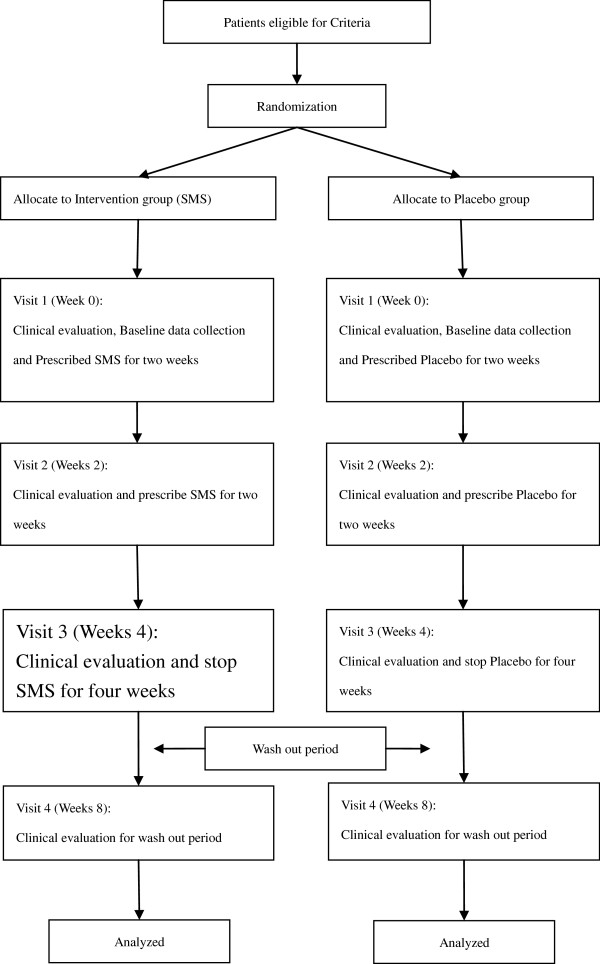
TCM therapeutic efficacy trial flow chart.

The study is conducted as a randomized, double-blind, placebo controlled trial to examine the effects of TCM in patients with cancer who are undergoing chemotherapy or radiotherapy. All participants are randomly assigned to an intervention or placebo group. The study procedures and informed consent form have been approved by the Institutional Review Board of Changhua Christian Hospital in Taiwan. (IRB no.070301). Figure 1 illustrates the flow diagram of the trial for both the intervention and placebo groups.

### Medication

According to the guidelines for clinical trials of new Chinese medicine announced by the Department of Health, Executive Yuan in Taiwan [15], only formulae recorded in ancient TCM books may be used for clinical trials in Taiwan. The formula employed in this trial, Shen-Mai-San (SMS) has a long history of application to disease treatment in China starting in the 12th century [16]. SMS is composed of three herb ingredients, namely *Ginseng radis*, *Liriope spicata*, and *Schizandrae fructus*. It was initially used for the treatment of a dual deficiency of qi and yin with symptoms of thirst and general weakness while suffering from heat stroke, fatigue, and so on [17]. According to TCM theory, SMS can reduce fatigue, thirst, general weakness, and so on. SMS is widely used now to treat patients with cardiac disease, fatigue and cancer [18,19]. Many patients have symptoms of general weakness, thirst, and fatigue after chemotherapy or radiotherapy. These symptoms are similar to the syndrome of qi and yin deficiency in TCM theory. In order to evaluate the efficacy of SMS in cancer patients after chemotherapy and radiotherapy, a randomized, double-blind, placebo-controlled trial will be performed.

SMS is manufactured as a concentrated herbal extract, packed 0.5 g per capsule, and labeled by Sun-Ten, a pharmaceutical company in Taiwan with good manufacturing practice (GMP). The placebo capsules are prepared with 0.5 g starch inside the same colored and sized capsules.

### Participants

Patients will be recruited from the outpatients of the Oncology and Chinese Medicine Department in ChangHua Christian Hospital, a medical center in central Taiwan. Sixty patients will be recruited for the study and divided into an intervention group and a placebo group.

### Inclusion criteria

In this study, inclusion criteria are as follows: (1) postoperative patients with histologically confirmed cancer within 3 years who are undergoing chemotherapy or radiotherapy; (2) more than 18 years old; (3) signed informed consent; (4) able to read Chinese; and (4) have the ability to take medicine by mouth.

### Exclusion criteria

Exclusion criteria include: (1) being pregnant; (2) breast feeding; (3) had completed chemotherapy or radiotherapy; (4) brain metastasis with Eastern Cooperative Oncology Group (ECOG) performance status of two to four; (5) suffering from delusion or hallucination; (6) acute infection; and (7) had received medications for other clinical trials.

### Randomization

Patients who meet the inclusion criteria will be randomly assigned to an intervention group (SMS) or placebo group by the clinical research coordinator (CRC).

### Blinding

The patients will receive capsules either with SMS or placebo. These capsules are the same in appearance, color and shape. The researchers and patients will not know which group the patients are allocated to from the appearance of the medication given.

### Intervention

One experienced TCM physician will be assigned to record the general data, TCM diagnostic data and prescribed medication for two weeks at the first visit. The patients will take the prescription to the pharmacy and the CRC will give either SMS or placebo in a capsule containing either 0.5 mg medication or starch. Patients will take eight capsules three times a day. The intervention period will be four weeks. Two weeks and four weeks after the first visit, patients will come back to out-patient-department (OPD) for clinical evaluation and medication.

After four weeks on the prescribed medication, the patients will stop taking SMS and placebo for another four weeks and then come back to OPD for clinical evaluation.

### Data collection

We will collect basic data such as gender, age, cancer type, stage, and metastasis or no metastasis at base line. Other than the basic data, such as gender and age, laboratory data, for example, WBC, Hb, Hct, CEA, platelet and HRV will be collected. GOT, GPT, BUN, and creatinine will also be recorded to facilitate the evaluation of liver and renal functions.

### Statistical analysis

Statistical analysis will be performed by the Department of Mathematics and the Institute of Statistics and Information Science, National Changhua University of Education in Taiwan. We will use the statistical software R to analyze the data. Paired-T test will be derived for statistically analysis.

### Primary outcome

Quality of life is evaluated multi-dimensionally using the Taiwan Chinese Version of the EORTC Quality of Life Questionnaire C30 (QLQ-C30). The patients will complete the questionnaire at baseline (first visit), two weeks later (second visit), four weeks later (third visit) and eight weeks later (fourth visit). The score will be calculated and compared within group and between groups before and after medication.

The laboratory data collected will be also used to evaluate the general health status and monitor the liver and renal functions to assess the safety of SMS.

## Discussion

There is an ever increasing number of patients suffering from cancer every year. Although various types of treatment are performed on a routine basis, surgery, chemotherapy and radiotherapy remain the major modes of cancer treatment. Through chemotherapy or radiotherapy, not only tumor cells, but also normal cells, are simultaneously killed. Many side effects, such as lower white blood cell count, general weakness and loss of appetite are often observed during and after the treatment. These side effects may lower the quality of life of these patients.

Many CAMs show significant improvements in chemotherapy- or radiotherapy-related side effects and TCM is the most common means of CAM in Taiwan. One study reported utilizing TCM to improve the quality of life in women undergoing chemotherapy for ovarian cancer and the result showed no significant difference in global health status but there was less neutropenia [13]. Another study used TCM for patients with colon or breast cancer requiring chemotherapy; there was no significant difference in hematologic toxicity but there was a significant impact on the control of nausea [20]. We think that we can select formulae according to the most common symptoms of the patients using TCM theory and lessen the side effects caused by chemotherapy in cancer treatment.

SMS is used for the treatment of myocardial infarction (MI) and has a protective effect for renal ischemic damage during MI [19,21]. Some studies also reported that SMS can improve sperm viability and sperm movement parameters *in vitro*[22]. According to ancient TCM theory, SMS could also prevent fatigue in the general population and especially when used in patients with qi and yin deficiency. It is composed of processed *G. radis, L. spicata, and S. fructus*. Ginseng was reported to have some activity and tolerable toxicity to cancer-related fatigue [23]. Ginseng polysaccharides also had anti-fatigue activity in a study in mice [24]. *L. spicata* was used for yin deficiency according to TCM theory and had hypoglycemic and hypolipidemic potential for type 2 diabetes[25]. *S. fructus* was used for the benefit of qi and promoted body fluid production according to TCM theory and was also reported to have effects in viral- and chemical-induced hepatitis [26].

All ingredients of SMS are considered to improve side-effects induced by chemotherapy or radiotherapy, as described above under TCM theory. In addition to the described effects of every ingredient of SMS, the formula also has a synergic effect not yet realized. Many studies have shown that TCM formulae exhibit more significant synergistic effects than all the ingredients combined [27,28]. Moreover, the safety of concentrated herbal extracts is also important in this study. Many studies have shown that herbs may cause liver or renal damage [29,30]. The concentrated herbal extracts used in this study are produced by a GMP pharmaceutical company in Taiwan. Due to the strict flow control during the manufacturing process, the concentrated herbal extracts are highly homogeneous with good quality control, which is very different from those obtained using traditional methods. Therefore, the concentrated herbal extracts are considered safer than the traditional herbs.

More efforts will be exerted to prove the efficacy of TCM in helping patients with cancer through ongoing studies. Future researchers will focus on finding effective TCM formulae to help patients ease the side effects of chemotherapy and radiotherapy.

## Trial status

At the time of manuscript submission, we have recruited thirty-six patients and are still recruiting patients.

## Abbreviations

BUN: Blood urea nitrogen; CAM: Complementary and alternative medicine; CEA: Carcinoembryonic antigen; CRC: Clinical research coordinator; ECOG: Eastern Cooperative Oncology Group; EORTC: European Organization for Research and Treatment of Cancer; GMP: Good manufacturing practice; Hb: Hemoglobin; Hct: Hematocrit; HRV: Heart rate variability; GOT: Glutamic-oxalacetic transaminase; GPT: Glutamic-pyruvic transaminase; OPD: Out-patient-department; QLQ-C30: Quality of Life Questionnaire C30; SMS: Shen-Mai-San; TCM: Traditional Chinese medicine.

## Competing interests

The authors declare that they have no competing interests.

## Authors’ contributions

CSC, LLC, CYC, STC, HCC and TCL all contributed to the development of the study protocol. LLC was the principal investigator and managed the protocol. CYC was involved in the initial draft of the manuscript and writing it. CSC and LLC were involved in reviewing the manuscript. All authors read and approved the final manuscript.
